# Pediatric Extremity Vascular Malformations: Diagnosis, Referral, and Limb Management from a Pediatric Orthopedic Perspective

**DOI:** 10.3390/jcm15103833

**Published:** 2026-05-15

**Authors:** Taichun Li, Jingmiao Wang, Hai Li, Ziming Zhang

**Affiliations:** Department of Pediatric Orthopedics, Shanghai Children’s Hospital, School of Medicine, Shanghai Jiao Tong University, No. 355 Luding Road, Shanghai 200062, China; litaichun@shchildren.com.cn (T.L.); wangjingmiao@shchildren.com.cn (J.W.); lihai@shchildren.com.cn (H.L.)

**Keywords:** vascular malformation, pediatric orthopedics, extremity, venous malformation, lymphatic malformation, arteriovenous malformation, fibro-adipose vascular anomaly, limb-length discrepancy, epiphysiodesis

## Abstract

Extremity vascular malformations in children and adolescents are congenital vascular developmental abnormalities that often present to pediatric orthopedic surgeons with pain, swelling, restricted motion, contracture, gait disturbance, limb asymmetry, and growth-related deformity rather than with an obvious vascular phenotype. The orthopedic importance of these lesions lies less in surface appearance than in their potential to affect muscle balance, joint integrity, osseous development, and peri-procedural safety. This review translates contemporary vascular anomaly classification and multidisciplinary management pathways into a practical orthopedic framework for diagnosis, referral, and longitudinal limb management. The most useful first step is to distinguish low-flow from high-flow lesions and then define lesion depth, periarticular or osseous involvement, coagulopathy risk, and syndromic overgrowth phenotype. Ultrasound is usually the first-line imaging modality for flow characterization, whereas magnetic resonance imaging is the cornerstone for defining extent and planning treatment. Plain radiographs remain highly relevant for identifying phleboliths, osseous remodeling, arthropathy, contracture-related deformity, and limb-length discrepancy. Venous malformations generally warrant pathway-based coagulation assessment, especially D-dimer and fibrinogen, because localized intravascular coagulopathy has direct implications for intervention and surgery. Arteriovenous malformations are best managed within specialist multidisciplinary teams. Fibro-adipose vascular anomaly and syndromic overgrowth phenotypes warrant particular attention because they frequently drive pain, contracture, and progressive limb imbalance. Outcome assessment in this field should extend beyond lesion size and incorporate pain, function, quality of life, and growth-related consequences. For pediatric orthopedic surgeons, management should move from late deformity correction toward early classification, early referral, longitudinal surveillance of joint and growth-related complications, and careful integration of local, surgical, and systemic therapies.

## 1. Introduction

Extremity vascular malformations are clinically important to pediatric orthopedic surgeons because they frequently manifest not as a straightforward vascular lesion but as chronic pain, recurrent swelling, restricted motion, contracture, hemarthrosis, gait disturbance, limb asymmetry, limb-length discrepancy (LLD), pelvic obliquity, and secondary spinal compensation [1,2]. Their orthopedic significance therefore lies less in surface appearance and more in the extent to which they affect muscle balance, joint integrity, osseous growth, and procedural safety.

Historically, orthopedic involvement has often occurred late, after major deformity or joint compromise had already developed. Contemporary vascular anomaly classification, specialist pathways, and multidisciplinary pediatric practice collectively support earlier orthopedic participation in risk stratification, musculoskeletal surveillance, and sequencing of interventional, surgical, and systemic therapy [3,4,5,6,7,8]. This shift is particularly relevant in syndromic or overgrowth phenotypes, in which musculoskeletal sequelae may be substantial. In a large KTS orthopedic series, most referred patients had clinically relevant limb inequality, and many ultimately required orthopedic procedures [9].

The objective of this review is to translate the current vascular anomaly framework into a practical diagnostic and management approach for pediatric orthopedic surgeons. Particular emphasis is placed on hemodynamic classification, lesion depth and tissue involvement, coagulation screening, growth-related consequences, referral thresholds, diagnostic safety issues, and the integration of image-guided, surgical, and medical therapies.

The need for an orthopedic-oriented review has also been emphasized, albeit indirectly, by prior literature describing the role of orthopedic surgeons in vascular malformations and associated syndromes. These reports highlight that hemarthrosis, contracture, osseous deformity, painful skeletal overgrowth, and limb hypertrophy are not secondary observations but central clinical problems in extremity disease. The present review therefore focuses not only on lesion classification, but also on how these lesions become musculoskeletal disorders requiring structured orthopedic surveillance and intervention planning [10].

## 2. Scope, Search Strategy, and Evidence Selection

This article was designed as a practical narrative review for pediatric orthopedic readers rather than as a formal systematic review. The objective was to synthesize classification frameworks, disease-specific vascular anomaly pathways, pediatric and multidisciplinary clinical literature, and relevant pediatric orthopedic evidence into an actionable framework for the diagnosis, referral, and longitudinal limb management of children with extremity vascular malformations. Therefore, PRISMA-based methodology, formal duplicate screening, and meta-analysis were not applied.

To improve transparency, a targeted literature search was performed in PubMed up to 1 March 2026. Search terms were combined according to topic and included “vascular malformation”, “vascular anomaly”, “extremity”, “pediatric orthopedics”, “venous malformation”, “lymphatic malformation”, “arteriovenous malformation”, “fibro-adipose vascular anomaly”, “Klippel-Trenaunay”, “Parkes Weber”, “capillary malformation arteriovenous malformation”, “PIK3CA-related overgrowth spectrum”, “limb length discrepancy”, “epiphysiodesis”, “localized intravascular coagulopathy”, “sirolimus”, “alpelisib”, and “trametinib”. Additional sources included official classification documents, VASCERN-VASCA patient pathways, GeneReviews chapters, the *Great Ormond Street Handbook of Paediatric Vascular Anomalies*, and reference lists of key reviews and disease-specific studies.

Sources were selected according to clinical relevance to pediatric extremity disease and according to a predefined evidence hierarchy. Priority was given to: (1) official classification documents and specialist pathways; (2) pediatric or multidisciplinary review articles directly relevant to vascular malformations; (3) disease-specific cohort studies, surgical series, and prospective therapeutic studies; (4) outcome-measurement studies relevant to vascular malformations; and (5) standard pediatric orthopedic reviews and consensus statements when vascular-malformation-specific orthopedic evidence was limited. Pediatric evidence was prioritized whenever available. Adult or mixed-age vascular anomaly literature was used when pediatric extremity-specific data were unavailable, particularly for outcome measurement and systemic therapy; such extrapolation is explicitly acknowledged where relevant.

This review focused on congenital vascular malformations of the extremities and their musculoskeletal consequences. Vascular tumors were not a primary focus, but selected tumor-related literature was included when relevant to diagnostic safety and differential diagnosis, particularly for painful, solid, or coagulopathic lesions. Articles were excluded from detailed discussion when they were unrelated to extremity disease, focused exclusively on adult nonorthopedic management without transferable relevance, or addressed technical interventions without implications for pediatric orthopedic decision-making.

Because high-level orthopedic evidence remains limited for LLD surveillance, growth modulation, and deformity correction in vascular malformations, standard pediatric orthopedic literature on epiphysiodesis and longitudinal follow-up was selectively incorporated [11,12,13,14]. In these instances, recommendations are framed as pragmatic orthopedic guidance rather than vascular-malformation-specific prospective evidence. Similarly, systemic and targeted therapy data are interpreted primarily in terms of symptom control, reduction in lesion activity, and facilitation of subsequent local or surgical treatment, because most available studies do not use orthopedic endpoints such as gait, contracture progression, joint preservation, LLD progression, or deformity correction as primary outcomes.

The evidence selection framework is summarized in Supplementary Table S1.

## 3. Classification and Terminology Relevant to Pediatric Orthopedic Practice

### 3.1. Vascular Tumors Versus Vascular Malformations

The first diagnostic distinction is between vascular tumors and vascular malformations. According to ISSVA, vascular tumors are proliferative lesions, whereas vascular malformations are congenital vascular developmental abnormalities [3,4]. This distinction is not merely semantic. A lesion with a proliferative course, substantial solid tissue, or consumptive coagulopathy should not automatically be assumed to be a malformation; kaposiform hemangioendothelioma remains an important differential diagnosis in painful or coagulopathic pediatric lesions [15].

### 3.2. Low-Flow Versus High-Flow

**Figure 1:**
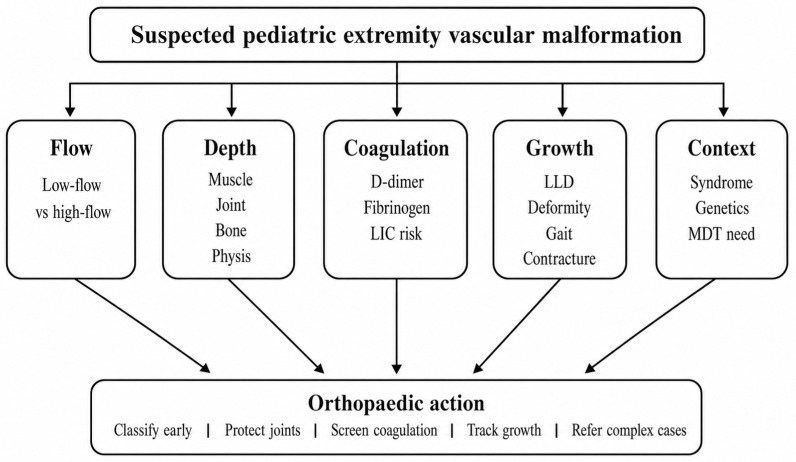
Five-domain orthopedic framework for pediatric extremity vascular malformations. The framework highlights flow type, tissue depth and musculoskeletal involvement, coagulation risk, growth-related consequences, and syndromic or molecular context. LIC, localized intravascular coagulopathy; LLD, limb-length discrepancy; MDT, multidisciplinary team.

For extremity lesions, the most useful first-line classification for pediatric orthopedic decision-making is hemodynamic: low-flow versus high-flow [3,4,16,17]. Low-flow lesions include venous malformations (VMs), lymphatic malformations (LMs), capillary malformations, and their combinations; high-flow lesions include arteriovenous malformations (AVMs) and arteriovenous fistulae. This distinction determines imaging escalation, urgency of referral, procedural risk, and treatment sequencing [16,18,19,20] (Figure 1).

### 3.3. A Five-Domain Orthopedic Framework

For clinical practice, five questions are often more useful than memorizing all ISSVA subtypes: (1) Is the lesion low-flow or high-flow? (2) Is it superficial, deep, periarticular, intra-articular, intraosseous, or physeal-related? (3) Is coagulopathy present? (4) Is there a syndromic or overgrowth phenotype? (5) What is the dominant orthopedic endpoint, namely pain, joint preservation, contracture prevention, growth control, or peri-procedural safety? This framework translates vascular-anomaly taxonomy into musculoskeletal decision-making.

## 4. Why Extremity Vascular Malformations Become Orthopedic Diseases

Extremity vascular malformations become orthopedic diseases when they involve tissues that determine limb mechanics and growth: subcutaneous tissue, muscle, tendon, periosteum, synovium, capsule, bone, and the physis [1,2]. Their burden includes not only pain and mass effect, but also muscle imbalance, recurrent hemarthrosis, cartilage injury, joint stiffness, contracture, gait asymmetry, LLD, circumferential asymmetry, and progressive deformity [1,21,22].

Intra-articular venous malformations of the knee are particularly important because they may present with recurrent unilateral knee pain, swelling, limited motion, and quadriceps wasting, while synovial involvement increases the risk of repeated hemarthrosis and secondary cartilage injury [21]. FAVA similarly has a high orthopedic impact because it is pain-dominant and function-dominant, often presenting with an intramuscular hard lesion and progressive contracture rather than a visibly vascular phenotype [23,24,25].

In syndromic settings, the musculoskeletal burden extends beyond a single region and affects overall limb growth and alignment [9,26,27,28,29]. Importantly, not all overgrowth phenotypes are hemodynamically equivalent. Low-flow dominant phenotypes such as KTS and many PROS-associated limb overgrowth presentations differ substantially from high-flow phenotypes such as Parkes–Weber/CM–AVM. This distinction has practical consequences for surgical risk, imaging escalation, and timing of orthopedic intervention.

The musculoskeletal phenotype profiles of the major lesion categories most relevant to pediatric orthopedic practice are summarized in Table 1.

## 5. Initial Orthopedic Assessment: Red Flags, Imaging, Coagulation, and Diagnostic Safety

### 5.1. History and Physical Examination

History should establish onset, tempo, growth-related progression, puberty-related change, response to trauma or infection, the presence of recurrent bleeding or oozing, monoarticular symptoms, and previous treatment response [6,16,17,18]. Examination should document lesion compressibility, skin discoloration, local warmth, thrill, bruit, joint motion, muscle wasting, contracture, gait, limb circumference, and apparent LLD. VM is usually soft, compressible, and nonpulsatile, whereas AVM more often presents with heat, bruit, or thrill and more rapid structural progression [16,17,20,30].

### 5.2. Red Flags for Early Specialist Referral

Red flags include suspected high-flow disease, deep or subfascial lesions, recurrent hemarthrosis, persistent monoarticular symptoms, rapidly progressive pain or contracture, marked limb asymmetry or overgrowth, abnormal coagulation tests, or the need for an invasive procedure in a lesion with venous or mixed components [7,8,16,19,31]. Local warmth, bruit, thrill, pulsatility, rapid progression, ulceration, or distal ischemic change should prompt high-flow imaging and referral to a vascular anomaly MDT before biopsy, excision, decompression, or other local orthopedic procedures.

### 5.3. Imaging Strategy

Ultrasound with Doppler is usually the first-line study for suspected extremity vascular malformations because it can characterize low-flow versus high-flow hemodynamics, identify cystic versus venous architecture, and suggest thrombosis or flow stasis [16,18,30]. MRI is the central imaging modality for deep lesions, intramuscular disease, periarticular involvement, intra-articular extension, osseous interface, and treatment planning [6,16,18,30,32]. CTA, MRA, and DSA are principally reserved for high-flow lesions or when vascular mapping is required before intervention [16,20,30,33].

Plain radiographs remain highly informative in pediatric orthopedic practice and should not be overlooked in a specialty increasingly dominated by cross-sectional imaging. They help identify phleboliths, osseous remodeling, cortical thinning, periarticular overgrowth, flexion deformity, joint damage, and LLD [6,7,9,11,13,14,21]. In growing children with asymmetry, standing long-limb imaging is especially useful for distinguishing structural LLD from pelvic obliquity, contracture, edema, or soft tissue hypertrophy [11,13,14].

### 5.4. Coagulation Assessment

Venous-component lesions should generally undergo coagulation screening, particularly when they are deep, extensive, symptomatic, or being considered for invasive treatment. The VASCERN-VASCA VM pathway recommends at minimum D-dimer and fibrinogen testing in all VM patients [7]. This is especially relevant in deep, extensive, multifocal, or syndromic disease because localized intravascular coagulopathy (LIC) may influence both spontaneous symptoms and peri-procedural risk [31,34,35]. For the pediatric orthopedic surgeon, coagulation assessment is therefore generally indicated before resection, arthroscopy, guided growth, or combined local intervention in lesions with a substantial venous component. However, these recommendations should be interpreted as pathway-based specialist guidance rather than as evidence derived from randomized comparative studies.

### 5.5. When to Suspect a Tumor and When to Consider Biopsy

Not every painful or enlarging vascular-appearing lesion is a malformation. Atypical or concerning features include a substantial solid component, unexpectedly rapid progression, discordance between the clinical course and typical malformation behavior, severe nocturnal pain, destructive osseous change, and coagulopathy out of proportion to lesion phenotype. In such settings, the possibility of a vascular tumor or a nonvascular neoplasm must be considered [15,16,18].

**Figure 2:**
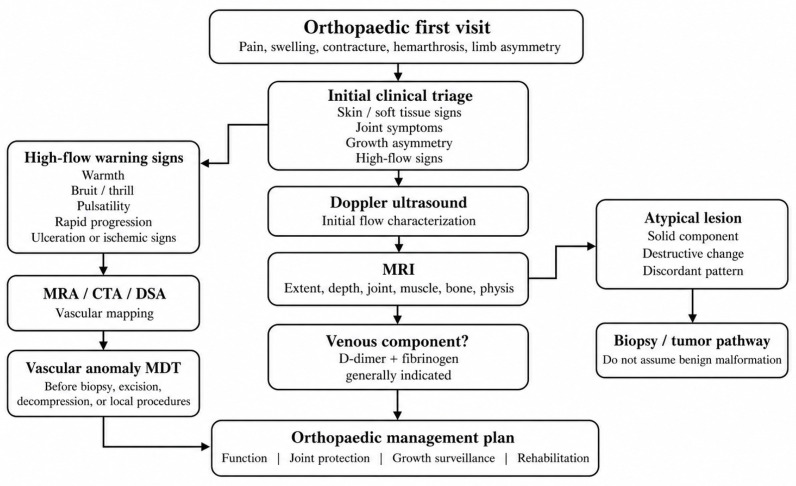
Diagnostic and referral algorithm for pediatric orthopedic surgeons evaluating suspected extremity vascular malformations. Doppler ultrasound is usually the first-line study for initial flow characterization, whereas MRI defines lesion extent and musculoskeletal relationships. High-flow warning signs should prompt vascular imaging and specialist referral before local orthopedic procedures. CTA, computed tomographic angiography; DSA, digital subtraction angiography; MDT, multidisciplinary team; MRA, magnetic resonance angiography; MRI, magnetic resonance imaging.

Biopsy is not routine for classic VM or LM, but it should be considered when the lesion does not fit the expected natural or radiological profile. In practical terms, the orthopedic surgeon should not proceed directly to vascular malformation management when malignancy or a vascular tumor remains plausible. Instead, the lesion should enter a dedicated tumor/biopsy pathway, ideally in coordination with radiology and the appropriate multidisciplinary team (Figure 2; Table 2).

## 6. Disease-Specific Considerations

### 6.1. Venous Malformations

A VM is the most common low-flow extremity vascular malformation and is closely associated with chronic pain, swelling, phlebolith formation, thrombosis, and activity-related symptoms [7,36,37]. The orthopedic phenotypes of greatest importance are deep intramuscular VMs, periarticular or intra-articular VMs, and extensive lesions complicated by LIC [7,21,22,31,34,35]. Management should be symptom- and function-driven. Compression is first-line supportive care; image-guided sclerotherapy is the principal local intervention; and surgery is mainly reserved for selected focal, periarticular, intra-articular, or residual functional lesions after interventional downstaging [6,7,36,37].

An intra-articular knee VM deserves particular orthopedic attention because recurrent hemarthrosis may drive irreversible cartilage damage and long-term loss of motion [21,22,38]. In a pediatric surgical series of intra-articular knee VMs, all children presented with pain and recurrent hemarthroses, preoperative chondropathy was already present in 63%, and the best outcomes were observed in well-demarcated lesions treated by complete resection [38]. Although diffuse lesions frequently persisted despite synovectomy, surgery reduced recurrent hemarthroses and appeared to protect the joint from further deterioration [38]. These observations support early specialist discussion of surgery in selected intra-articular lesions, particularly before established chondropathy and fixed functional impairment.

### 6.2. Lymphatic Malformations

An LM is categorized as macrocystic, microcystic, or mixed [8,30,32]. Macrocystic lesions more often behave as compressive cystic masses and respond better to sclerotherapy, whereas microcystic disease is more infiltrative and is frequently associated with inflammation, oozing, bleeding, pain, and diffuse soft tissue burden [8,32]. In the limbs, an LM should be approached as a chronic soft tissue load that may impair motion, promote contracture, and complicate gait and footwear rather than simply as a cystic lesion. This distinction is relevant to orthopedic planning because the main burden may be joint limitation and soft tissue intolerance rather than lesion volume alone.

### 6.3. Arteriovenous Malformations

An AVM is defined by a nidus connecting arterial inflow and venous outflow without a normal capillary bed [3,19,20]. The orthopedic pitfall is to misinterpret an AVM as a localized soft tissue lesion amenable to simple excision. In reality, incomplete proximal control fails because the nidus recruits collateral inflow and redistributes hemodynamic burden [20]. All suspected or confirmed AVMs should therefore be managed within specialist multidisciplinary pathways [19]. The orthopedic role is to identify high-flow red flags, define the musculoskeletal burden, and contribute to joint-preserving and limb-preserving strategy rather than to undertake isolated local surgery.

### 6.4. Fibro-Adipose Vascular Anomaly

A FAVA is highly relevant to pediatric orthopedic surgeons because it is pain-dominant and function-dominant [23,24,25]. Typical features include persistent pain, a hard intramuscular lesion, progressive contracture, and substantial functional limitation. Repeated sclerotherapy alone often does not address the primary orthopedic complaint. Cryoablation, selective debulking, contracture release, and rehabilitation should therefore be sequenced according to the dominant functional problem [23,24]. In practice, FAVA and periarticular or intra-articular VM should be considered orthopedic priority lesions because delayed recognition may lead to irreversible joint or muscle dysfunction.

### 6.5. Low-Flow Overgrowth Phenotypes

KTS and many PROS-related limb phenotypes are best interpreted as low-flow dominant overgrowth disorders with orthopedic implications extending beyond the vascular lesion itself [9,26,27,29,39]. These patients may present with toe enlargement, circumferential asymmetry, LLD, pelvic obliquity, gait disturbance, patellofemoral imbalance, and progressive deformity. The orthopedic task is longitudinal growth surveillance and deformity monitoring, with attention to both structural bone length and soft tissue burden. Because repeated local procedures rarely eliminate the biological tendency toward overgrowth or recurrence, longitudinal management is often more important than any single intervention [10,39].

### 6.6. High-Flow Overgrowth Phenotypes

Parkes–Weber/capillary malformation–arteriovenous malformation syndrome (CM–AVM) phenotypes differ fundamentally from KTS because they arise in a high-flow hemodynamic context [28,29]. Limb overgrowth in this setting must be interpreted alongside the risk of persistent arteriovenous shunting, tissue overload, and the greater hazard of unsupervised local surgery. These patients often require earlier vascular imaging escalation, more urgent specialist referral, and closer coordination between orthopedic and vascular anomaly teams than low-flow overgrowth phenotypes.

## 7. Treatment Principles and Orthopedic Decision-Making

### 7.1. Treatment Goals

The primary goals of management are symptom control, preservation of joint function, maintenance of muscle balance and range of motion, prevention or mitigation of growth-related deformity, minimization of peri-procedural risk, and improvement in quality of life [1,5,6,7,8,19]. Complete eradication of the lesion is not always realistic and should not supersede functional decision-making.

### 7.2. Conservative and Rehabilitative Management

Compression garments, physical therapy, orthoses, shoe lifts, gait training, and pain-directed supportive care form the basis of management for many patients [6,7,8]. Rehabilitation should be introduced early rather than after contracture or major asymmetry is established.

### 7.3. Image-Guided Local Therapy and Surgery

For low-flow lesions, local treatment is usually based on image-guided sclerotherapy [30,32,33,36,37]. Orthopedic surgeons should view sclerotherapy as one element within a functional treatment sequence rather than as a competing specialty procedure. Surgery is most relevant when the lesion is focal, functionally dominant, periarticular, intra-articular, or associated with fixed contracture or biomechanical imbalance [1,6,21,23,24,36]. For AVM, surgery without hemodynamic planning may be harmful [19,20].

### 7.4. Limb-Length Discrepancy and Growth Modulation

LLD management in children with vascular malformations is challenging because lesion-specific high-level evidence is scarce. Orthopedic planning should therefore combine lesion-specific assessment with established pediatric orthopedic principles [1,9,11,12,13,14].

The first step is to distinguish true structural discrepancy from functional asymmetry caused by pelvic obliquity, edema, soft tissue hypertrophy, or joint contracture [6,9,13,14]. Once structural discrepancy is confirmed, serial prediction rather than single-time-point assessment is required.

In general pediatric orthopedic practice, predicted mature LLD of less than 2 cm is usually managed conservatively, 2–5 cm is the principal range for considering epiphysiodesis if sufficient growth remains, and discrepancies greater than 5 cm usually prompt consideration of lengthening or combined strategies [11,12,13,14]. However, these thresholds should be interpreted as pragmatic orthopedic guidance rather than vascular-malformation-specific prospective evidence. In patients with vascular malformations, treatment decisions must also account for lesion biology, soft tissue burden, contracture, gait asymmetry, overgrowth phenotype, and the broader multidisciplinary treatment sequence.

The lower-limb physes most commonly selected for epiphysiodesis are the distal femur and proximal tibia because of their accessibility and growth contribution [11,12,14]. As a practical approximation, residual annual growth may be considered roughly 0.95 cm at the distal femur and 0.64 cm at the proximal tibia [12,14]. The current pediatric orthopedic literature suggests that epiphysiodesis is the treatment of choice for predicted mature discrepancies of 2–5 cm when the physes remain open and sufficient growth remains [11,12]. Nevertheless, timing remains imperfect, and at least two growth-prediction methods should be used before definitive epiphysiodesis planning [11,12,13]. Consensus-based follow-up recommendations support baseline full-length lower-limb radiography, repeated prediction of mature discrepancy and the timing of epiphysiodesis, annual reassessment of lift height before puberty, and 6-monthly reassessment during puberty [13]. In children whose current and predicted discrepancy remains below 2 cm, radiographic follow-up every 2–3 years before puberty is generally sufficient, depending on etiology and progression [13]. These follow-up intervals should be individualized in vascular malformation patients according to lesion behavior and associated soft tissue or vascular burden.

**Figure 3:**
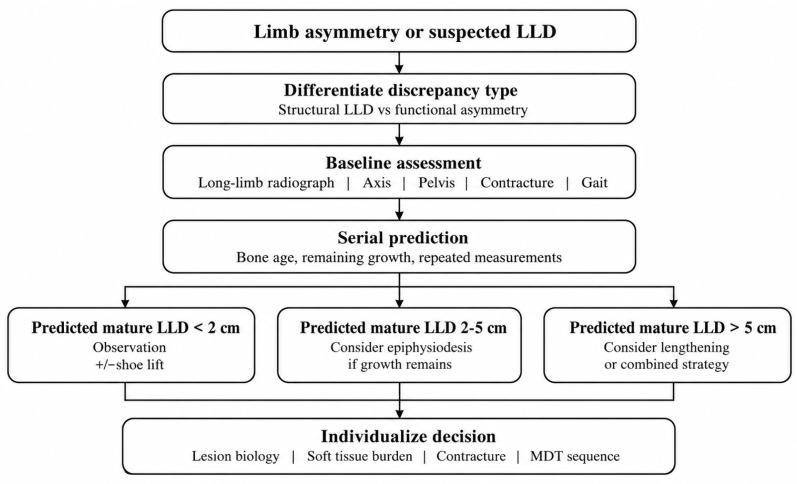
Longitudinal LLD and growth-management pathway in children with extremity vascular malformations. Structural LLD should be distinguished from functional asymmetry. The thresholds shown are pragmatic extrapolations from general pediatric orthopedic practice rather than vascular-malformation-specific prospective thresholds. LLD, limb-length discrepancy; MDT, multidisciplinary team.

The choice of technique should also be framed cautiously. Permanent percutaneous epiphysiodesis and percutaneous epiphysiodesis using transphyseal screws (PETS) remain the best-established approaches for LLD management [11,12]. By contrast, guided growth constructs are used more commonly for reversible physeal tethering or angular correction and should not be presented as clearly superior for definitive LLD equalization [12]. Comparative data do not demonstrate clear superiority of one permanent technique over another [11]. Accordingly, the decision to proceed with growth modulation should be individualized and understood as elective rather than automatic (Figure 3; Table 3).

### 7.5. Peri-Procedural Hemostatic Management

VM and combined slow-flow lesions with venous components may harbor LIC [7,31,34,35]. The practical implication is that D-dimer and fibrinogen should generally be incorporated into procedural planning for surgery, arthroscopy, guided growth, deformity correction, and combined interventions. Standard coagulation tests alone do not sufficiently characterize risk in this setting. Abnormal D-dimer, low fibrinogen, thrombocytopenia, prior thrombosis, or a clinically significant bleeding history should generally prompt hematology consultation before arthroscopy, resection, epiphysiodesis, osteotomy, or combined vascular–orthopedic procedures.

### 7.6. Systemic and Targeted Therapy

Systemic therapy has become an important adjunct in complex vascular malformations. For the pediatric orthopedic surgeon, its relevance lies in symptom control, reduction in lesion activity, facilitation of later intervention, and possible modification of disease trajectory. Current systemic and targeted therapy studies rarely use orthopedic endpoints such as gait, contracture progression, joint preservation, LLD progression, or deformity correction as primary outcomes. Therefore, their relevance to orthopedic practice should be interpreted primarily in terms of symptom control, reduction in lesion activity, and facilitation of subsequent local or surgical treatment.

The strongest current evidence supports sirolimus in selected complicated slow-flow malformations [40,41,42]. Importantly, the therapeutic signal is not uniform across diagnostic groups. In the phase II prospective study by Adams et al., no complete radiologic responses were observed; most evaluable patients achieved partial responses, and significant toxicities included grade ≥ 3 blood/bone marrow toxicity in 27% of cases [40]. The PERFORMUS trial did not show an overall significant reduction in lesion volume across all slow-flow malformations, but did show a significant MRI-based volumetric decrease in pure lymphatic malformations. It also suggested that symptom benefit—particularly pain, bleeding, and oozing—was more pronounced in combined malformations, whereas benefits in pure venous malformations were comparatively more limited and symptom-based rather than volumetric [41]. The VASE phase III study further showed clinical improvement in 85% of patients, often within the first month, with grade 3–4 adverse events in 18%; sirolimus increased the feasibility of surgery or sclerotherapy in 15% of initially ineligible patients, but symptom recurrence after discontinuation remained common [42]. This interpretation is also consistent with broader literature syntheses, which suggest overall benefit but emphasize that the sirolimus evidence base remains dominated by heterogeneous lower-level reports outside the few prospective studies now available [43]. In addition, retrospective hematology data suggest that some of the symptomatic benefit of sirolimus may parallel improvement in venous-component coagulopathy, particularly reduction in D-dimer levels in slow-flow lesions [44]. Collectively, these data support sirolimus mainly as a disease-control and bridge-to-intervention therapy, particularly in complicated LM and combined slow-flow malformations, rather than as a universal alternative to local treatment or a curative option.

Targeted therapy is increasingly relevant for genetically defined and syndromic disease. In PIK3CA-related overgrowth spectrum, alpelisib provides real-world evidence of benefit in molecularly selected patients: in EPIK-P1, 37.5% of evaluable complete cases achieved a ≥20% reduction in target lesion volume at 24 weeks, while additional clinical benefit independent of lesion-volume reduction was observed across the full study population [39]. These data support PI3Kα inhibition as a rational therapeutic strategy in molecularly selected PROS requiring systemic therapy. However, EPIK-P1 was a retrospective, non-interventional study without a control arm, and lesion-specific response could not be fully evaluated [39]. For AVM, genotype-directed targeted therapy remains promising but still early in its evidence base. In a KRAS-mutant metameric AVM, trametinib treatment was associated with a greater than 75% reduction in arterial inflow after 6 months, monitored by phase-contrast magnetic resonance angiography, illustrating that hemodynamic biomarkers may be more informative than lesion size alone in selected high-flow disease [45]. At present, such approaches should be interpreted as emerging targeted therapy rather than established standard care. This broader interpretation is also supported by contemporary translational reviews of vascular-anomaly biology and targeted treatment, as well as by the regulatory summary supporting alpelisib in PROS [46,47,48]. Earlier pediatric genotype-guided experience likewise supports a precision-medicine approach in selected arteriovenous malformations [49].

For orthopedic practice, the key question is therefore not simply which drug is available, but when molecular diagnosis should be pursued—particularly in recurrent disease, overgrowth phenotypes, refractory lesions, or clinical behavior that is inconsistent with conventional classification (Table 4).

Because orthopedic procedures in children with extremity vascular malformations may involve hemodynamic, coagulation, diagnostic, and growth-related risks that are not encountered in routine pediatric orthopedic practice, preoperative planning should be structured and multidisciplinary. Table 5 summarizes a practical checklist for pediatric orthopedic intervention in suspected or confirmed extremity vascular malformations. The checklist is not intended to replace local vascular anomaly team protocols, but to ensure that flow status, venous coagulopathy risk, AVM warning signs, deep venous anatomy, diagnostic safety, growth assessment, and rehabilitation planning are considered before surgery.

## 8. Multidisciplinary Referral and Longitudinal Surveillance

Extremity vascular malformations should be managed longitudinally rather than episodically. The threshold for multidisciplinary referral should be low when lesions are deep, extensive, symptomatic, high-flow, syndromic, or associated with coagulopathy [7,8,16,19,20,31]. Orthopedic surgeons should collaborate closely with interventional radiology, hematology, genetics, rehabilitation, and, when appropriate, pediatric surgery or plastic surgery.

Longitudinal surveillance should document pain pattern, range of motion, contracture, muscle function, gait, limb circumference, LLD, axial alignment, pelvic balance, and radiographic progression [1,6,9,11,13,14]. During puberty and rapid growth, follow-up intensity should generally increase because discrepancy and functional imbalance may progress more rapidly.

## 9. Discussion

A pediatric orthopedic review of extremity vascular malformations should not stop at lesion classification. Its clinical value lies in reframing vascular-anomaly knowledge in terms of joint preservation, function, growth disturbance, and procedural safety. Earlier orthopedic literature already recognized that vascular malformations and associated syndromes may present with hemarthrosis, contracture, osseous deformity, painful skeletal overgrowth, and limb hypertrophy, thereby requiring structured orthopedic participation within multidisciplinary teams [10]. That observation remains highly relevant today.

Several practical recommendations in this review arise from different levels of evidence and should be interpreted accordingly. Hemodynamic classification, the importance of MRI for deep or periarticular disease, and the musculoskeletal consequences of intra-articular or intramuscular lesions are supported by classification papers, specialist pathways, multidisciplinary reviews, and lesion-specific series [3,4,5,7,8,10,16,18,19,21,22,38]. By contrast, thresholds for LLD surveillance, timing of epiphysiodesis, and the selection of growth-modulation techniques are not specific to vascular malformations and remain extrapolated from standard pediatric orthopedic literature [11,12,13,14]. This distinction is essential to avoid presenting pragmatic orthopedic practice as lesion-specific high-level evidence.

Outcome measurement remains a major limitation in this field. A systematic review and meta-analysis reported that patients with vascular malformations have poorer health-related quality of life than the general population, particularly in the domains of bodily pain and mental health [50]. The OVAMA project established that core outcome domains in peripheral vascular malformations should include radiological assessment, physician-assessed location-specific signs, patient-reported pain, symptom severity, quality of life, satisfaction, and adverse events [51]. This is directly relevant to orthopedic research because current studies still frequently overemphasize lesion size while underreporting gait, contracture progression, limb asymmetry, patient-reported function, and quality of life. Most quality-of-life studies also aggregate heterogeneous lesion phenotypes and are not tailored to pediatric musculoskeletal endpoints. Moreover, the OVAMA responsiveness study suggested that generic instruments such as SF-36 and Skindex-29 are insufficiently responsive to change in this patient population, indicating that vascular-malformation-specific patient-reported outcome measures remain needed [52]. Because the responsiveness study was conducted in adults with peripheral vascular malformations, direct extrapolation to pediatric orthopedic populations should therefore be cautious. Future orthopedic studies should therefore align more closely with this core-outcome framework and integrate musculoskeletal and patient-centered endpoints alongside lesion-centered imaging data.

Diagnostic safety also deserves emphasis. Not every painful or enlarging vascular-appearing lesion is a malformation, and the orthopedic pathway must include a mechanism for redirecting atypical lesions to biopsy or tumor work-up rather than persisting with the assumption of a benign vascular malformation. This is particularly important for deep painful lesions, lesions with substantial solid tissue, unexpectedly destructive osseous change, or coagulopathy out of proportion to lesion phenotype [15,16,18].

Systemic therapy should also be interpreted through an orthopedic lens. The principal value of sirolimus, alpelisib, or emerging targeted therapies is not simply lesion shrinkage, but symptom reduction, improved function, reduced soft tissue burden, and improved feasibility of later intervention. In this sense, orthopedic surgeons should understand systemic therapy as part of a longitudinal limb-management strategy rather than as a competing nonoperative alternative.

### Key Controversies and Evidence Gaps

Several clinically important controversies remain unresolved. First, thresholds for LLD surveillance and epiphysiodesis are extrapolated from general pediatric orthopedic practice rather than vascular-malformation-specific prospective evidence. Second, the optimal sequencing of orthopedic surgery relative to sclerotherapy, embolization, and systemic therapy remains poorly defined. Third, intra-articular VM and FAVA are orthopedic-priority lesions, but available evidence remains largely limited to small series and expert experience. Fourth, systemic and targeted therapies may reduce symptoms and lesion activity, but their long-term effects on skeletal growth, deformity progression, and orthopedic outcomes remain uncertain. Future studies should integrate gait, range of motion, contracture progression, joint preservation, LLD progression, patient-reported outcomes, and quality of life into vascular anomaly research.

## 10. Conclusions

From a pediatric orthopedic perspective, extremity vascular malformations are important because they influence muscle function, joint integrity, skeletal development, and peri-procedural safety. Their management should begin with correct hemodynamic classification, careful assessment of musculoskeletal involvement, appropriate coagulation screening in venous-component lesions, and early recognition of syndromic overgrowth phenotypes. Orthopedic care should move from late deformity correction toward early classification, early referral, and longitudinal limb surveillance. A practical framework centered on flow type, function, coagulation, growth, and molecular context is more useful for orthopedic decision-making than lesion nomenclature alone.

## Figures and Tables

**Table 1:** Musculoskeletal phenotype profile of major pediatric extremity vascular malformations relevant to orthopedic practice.

Lesion Type	Flow	Typical Extremity Presentation	Principal Orthopedic Concerns	Key Initial Orthopedic Actions
Venous malformation (VM)	Low	Pain, swelling, positional fullness, phleboliths, intralesional thrombosis	Deep intramuscular pain, periarticular or intra-articular disease, hemarthrosis, joint dysfunction, occasional LLD in complex disease	Ultrasound and MRI; D-dimer and fibrinogen assessment is generally indicated, particularly if lesions are deep, extensive, symptomatic, or procedural; early specialist referral if deep or extensive
Lymphatic malformation (LM)	Low	Cystic or infiltrative soft tissue lesion, inflammation, oozing, bleeding	Soft tissue burden, reduced range of motion, contracture, gait limitation	Ultrasound and MRI; early referral if diffuse, infiltrative, or function limiting
Arteriovenous malformation (AVM)	High	Heat, bruit, thrill, pulsatility, progressive enlargement, ulceration	Pain, tissue overload, progressive deformity, substantial risk with unsupervised local surgery	Treat as a high-flow lesion; escalate to MRI/MRA or CTA/DSA and refer immediately to MDT
Fibro-adipose vascular anomaly (FAVA)	Low	Persistent pain, hard intramuscular lesion, progressive contracture	Severe functional limitation, muscle imbalance, progressive contracture	MRI-focused work-up; early specialist referral and contracture-oriented planning
Low-flow dominant overgrowth phenotypes (e.g., KTS, selected PROS)	Low-flow dominant	Limb overgrowth, toe enlargement, circumferential asymmetry, venous or lymphatic burden	LLD, pelvic obliquity, gait disturbance, deformity progression, patellofemoral imbalance	Baseline long-limb radiographs plus lesion imaging; longitudinal MDT follow-up and growth surveillance
High-flow dominant overgrowth phenotypes (e.g., Parkes–Weber/CM–AVM)	High-flow dominant	Limb overgrowth with capillary lesions and fast-flow shunting	LLD, tissue overload, progressive deformity, major risk with unsupervised local surgery	Immediate flow-directed imaging escalation and specialist MDT referral; orthopedic follow-up integrated with vascular planning

**Table 2:** Actionable decision points for pediatric orthopedic surgeons evaluating suspected extremity vascular malformations.

Clinical Scenario	Suggested Action	Orthopedic Rationale	Evidence Basis
Uncertain vascular lesion at first presentation	Perform Doppler ultrasound as the initial flow-characterization study.	Distinguishes low-flow from high-flow lesions and guides further imaging.	Review-based/imaging-algorithm evidence
Deep, subfascial, periarticular, intra-articular, intramuscular, osseous, or physeal-related lesion	Obtain MRI to define lesion extent and musculoskeletal relationships.	Defines operative risk, joint involvement, muscle involvement, and growth-related anatomy.	Review-based/specialist practice
Lesion > 5 cm, uncertain flow type, substantial solid component, or suspected high-flow lesion	Escalate to contrast-enhanced MRI, MRA, CTA, or DSA as appropriate.	Avoids misclassification and improves vascular mapping before intervention.	Diagnostic-algorithm evidence
Local warmth, bruit, thrill, pulsatility, rapid progression, ulceration, or distal ischemic change	Treat as suspected high-flow disease and refer to a vascular anomaly MDT before biopsy, excision, decompression, or other local procedures.	Prevents inappropriate local surgery in possible AVM.	Pathway/expert consensus
Venous-component lesion, especially if deep, extensive, symptomatic, syndromic, or planned for invasive treatment	Assess D-dimer and fibrinogen as part of pathway-supported coagulation screening.	Identifies LIC and informs procedural bleeding/thrombotic risk.	Specialist pathway/hematology review
Recurrent monoarticular swelling, hemarthrosis, unexplained quadriceps wasting, or persistent knee pain	Evaluate for periarticular or intra-articular VM using MRI and specialist referral.	Early recognition may reduce recurrent hemarthrosis and joint damage.	Case series/cohort evidence
Persistent pain with intramuscular firm lesion and contracture	Consider FAVA and obtain MRI; refer for multidisciplinary functional planning.	FAVA may require cryoablation, debulking, contracture release, and rehabilitation rather than repeated sclerotherapy alone.	Disease-specific review/case-series evidence
Limb overgrowth, toe enlargement, circumferential asymmetry, pelvic obliquity, or progressive LLD	Assess for syndromic overgrowth and initiate longitudinal orthopedic surveillance.	Tracks growth-related deformity and distinguishes structural from functional discrepancy.	Cohort evidence/orthopedic extrapolation
Atypical solid lesion, destructive osseous change, severe night pain, or clinical-radiologic discordance	Redirect to biopsy or tumor pathway rather than routine malformation management.	Prevents misdiagnosis of vascular tumors or nonvascular neoplasms.	Diagnostic safety principle/expert practice

*Abbreviations*: AVM, arteriovenous malformation; CTA, computed tomographic angiography; DSA, digital subtraction angiography; FAVA, fibro-adipose vascular anomaly; LIC, localized intravascular coagulopathy; LLD, limb-length discrepancy; MDT, multidisciplinary team; MRA, magnetic resonance angiography; MRI, magnetic resonance imaging; VM, venous malformation. Evidence-basis categories are intended to clarify the strength and origin of practical recommendations; they should not be interpreted as formal guideline grades.

**Table 3:** Orthopedic surveillance and intervention framework for limb-length discrepancy and growth-related deformity.

Clinical Stage	Suggested Assessment	Imaging Interval	Main Orthopedic Goal	Preferred Strategy
Suspected asymmetry, early follow-up	Clinical block test, gait, range of motion, pelvic level, baseline long-limb imaging if structural inequality suspected	Baseline	Separate true structural LLD from functional asymmetry	Observation, treat contracture or soft tissue issues, consider shoe lift if symptomatic
Predicted mature LLD < 2 cm	Repeat clinical measurement, reassess growth pattern and symptoms	Every 2–3 years before puberty if stable	Maintain function and monitor progression	Conservative management, shoe compensation as needed
Predicted mature LLD 2–5 cm with growth remaining	Serial growth prediction using at least two methods, bone age or pubertal assessment when needed	Closer interval during growth, typically annual and every 6 months during puberty	Time growth modulation appropriately	Plan epiphysiodesis if overall disease context is suitable
Predicted mature LLD > 5 cm or major deformity burden	Full deformity work-up, standing radiographs, soft tissue and lesion burden assessment	Individualized, generally closer follow-up	Prepare staged reconstruction strategy	Lengthening or combined strategy; epiphysiodesis may still be adjunctive
Post-growth-modulation or post-intervention	Clinical alignment, gait, range of motion, repeat radiography	Every 4–6 months when growth modulation is active, then individualized	Detect undercorrection, overcorrection, or secondary axis deviation	Modify shoe lift, observe, or plan further intervention as needed

*Note*: These thresholds are extrapolated from general pediatric orthopedic practice and should not be interpreted as vascular-malformation-specific prospective thresholds.

**Table 4:** Systemic and targeted therapies relevant to pediatric orthopedic practice in extremity vascular malformations.

Therapy	Main Pathway	Most Relevant Lesions	Orthopedic Relevance	Key Limitation	Evidence Basis
Sirolimus	mTOR inhibition	Complicated slow-flow malformations, especially LM and combined slow-flow malformations	May reduce pain, oozing, bleeding, swelling, and lesion activity; may facilitate later sclerotherapy or surgery.	Not curative; volumetric benefit is variable; recurrence after discontinuation may occur; adverse events require monitoring.	Prospective phase II/III and randomized data; systematic-review evidence
Sirolimus in LIC-associated slow-flow lesions	mTOR inhibition	Slow-flow lesions with venous components and coagulopathy	May improve D-dimer and coagulopathy-associated symptoms in selected patients.	Evidence remains limited and mostly retrospective; not a substitute for peri-procedural hematology planning.	Retrospective cohort evidence
Alpelisib	PI3Kα inhibition	PIK3CA-related overgrowth spectrum and selected PIK3CA-related vascular overgrowth phenotypes	May reduce overgrowth-related lesion burden and improve symptoms in molecularly selected patients.	EPIK-P1 was retrospective and non-interventional without a control arm; lesion-specific response could not be fully evaluated.	Molecularly selected real-world cohort data; regulatory-approval framework
Trametinib and related MEK inhibitors	RAS/MAPK pathway inhibition	Selected AVM with MAPK-pathway variants	May reduce high-flow lesion activity and hemodynamic burden in selected genotype-defined cases.	Evidence remains case-based or small-series; long-term pediatric skeletal and orthopedic outcomes are unknown.	Emerging case-based/genotype-guided evidence
Other targeted approaches	Pathway-specific	Selected genetically defined vascular malformations or overgrowth syndromes	Potential role in refractory, recurrent, or syndromic lesions where conventional local therapy is insufficient.	Requires specialist molecular diagnosis; long-term safety and orthopedic endpoints remain incompletely defined.	Emerging translational/ specialist-center evidence

*Abbreviations*: AVM, arteriovenous malformation; LIC, localized intravascular coagulopathy; LM, lymphatic malformation; MAPK, mitogen-activated protein kinase; MEK, mitogen-activated protein kinase kinase; mTOR, mammalian target of rapamycin; PI3K, phosphoinositide 3-kinase. Current systemic and targeted therapy studies rarely use orthopedic endpoints such as gait, contracture progression, joint preservation, limb-length discrepancy progression, or deformity correction as primary outcomes. Therefore, orthopedic relevance should be interpreted mainly in terms of symptom control, reduction in lesion activity, and facilitation of subsequent local or surgical treatment.

**Table 5:** Preoperative checklist for pediatric orthopedic intervention in suspected or confirmed extremity vascular malformations.

Domain	Checklist Item	Orthopedic Relevance
Flow status	Has low-flow versus high-flow status been established by clinical assessment and Doppler ultrasound, with further vascular imaging when needed?	Avoids inappropriate local surgery in high-flow lesions.
AVM exclusion	Are local warmth, bruit, thrill, pulsatility, rapid progression, ulceration, or distal ischemic signs present?	Suspected AVM should prompt vascular imaging and MDT referral before biopsy, excision, decompression, or other local procedures.
Anatomic extent	Has MRI defined intramuscular, periarticular, intra-articular, osseous, physeal, or neurovascular involvement?	Determines operative risk, surgical target, and functional endpoint.
Venous component	If a significant venous component is present, have D-dimer and fibrinogen been assessed as part of pathway-supported coagulation screening?	Identifies possible LIC before arthroscopy, resection, epiphysiodesis, osteotomy, or combined procedures.
Hematology input	Is hematology consultation needed for abnormal D-dimer, low fibrinogen, thrombocytopenia, prior thrombosis, bleeding history, or extensive VM?	Reduces peri-procedural bleeding and thrombotic risk.
Deep venous anatomy	Has deep venous drainage been reviewed when relevant?	Avoids compromising essential venous outflow or increasing thrombotic risk.
MDT review	Has the case been discussed with a vascular anomaly MDT for AVM, extensive lesions, syndromic disease, recurrent lesions, or planned combined treatment?	Ensures appropriate sequencing of embolization, sclerotherapy, systemic therapy, and orthopedic intervention.
Diagnostic safety	Is the lesion typical of a malformation, or are there solid, aggressive, destructive, or discordant features requiring biopsy or tumor-pathway evaluation?	Prevents misdiagnosis of vascular tumors or nonvascular neoplasms.
Growth and alignment	Have LLD, mechanical axis, pelvic balance, contracture, and remaining growth been assessed?	Guides growth modulation, deformity correction, and timing of intervention.
Surgical endpoint	Is the goal clearly defined: biopsy, joint preservation, hemarthrosis control, contracture release, debulking, growth modulation, deformity correction, or pain reduction?	Prevents poorly targeted surgery and clarifies expected benefit.
Rehabilitation plan	Is postoperative rehabilitation planned, including range-of-motion protection, strengthening, gait training, orthotic support, or compression when appropriate?	Protects joint motion, gait, and long-term function.

*Abbreviations*: AVM, arteriovenous malformation; LIC, localized intravascular coagulopathy; LLD, limb-length discrepancy; MDT, multidisciplinary team; MRI, magnetic resonance imaging; VM, venous malformation. This checklist is intended as a practical peri-procedural safety framework and should be adapted to local vascular anomaly team protocols.

## Data Availability

No new data were created or analyzed in this study.

## Supplementary Materials

The following supporting information can be downloaded at: https://www.mdpi.com/article/10.3390/jcm15103833/s1, The search strategy and evidence selection framework are provided in Supplementary Table S1.

## Abbreviations

The following abbreviations are used in this manuscript:

AVM	arteriovenous malformation
CBC	complete blood count
CM–AVM	capillary malformation–arteriovenous malformation syndrome
CTA	computed tomographic angiography
DSA	digital subtraction angiography
FAVA	fibro-adipose vascular anomaly
ISSVA	International Society for the Study of Vascular Anomalies
KHE	kaposiform hemangioendothelioma
KTS	Klippel–Trenaunay syndrome
LIC	localized intravascular coagulopathy
LLD	limb-length discrepancy
LM	lymphatic malformation
MDT	multidisciplinary team
MRA	magnetic resonance angiography
MRI	magnetic resonance imaging
mTOR	mammalian target of rapamycin
PETS	percutaneous epiphysiodesis using transphyseal screws
PI3K	phosphatidylinositol 3-kinase
PROS	PIK3CA-related overgrowth spectrum
QoL	quality of life
US	ultrasound
VM	venous malformation
