# Supplementary material for: Pediatric Extremity Vascular Malformations: Diagnosis, Referral, and Limb Management from a Pediatric Orthopedic Perspective
Source: J Clin Med. 2026 May 15;15(10):3833. doi: 10.3390/jcm15103833 (PMC13207326; doi:10.3390/jcm15103833)
Supplement: Supplementary file 1 [file jcm-15-03833-s001.zip › jcm-4284206-supplementary.pdf]

Supplementary Table S1. Search strategy and evidence selection framework.

**Domain**	**Approach used in this review**
Review type	Practical narrative review focused on pediatric orthopaedic decision-making. The review was not designed as a systematic review, and PRISMA-based methodology was not applied.
Main database	PubMed.
Search date	Literature searched up to 1 March 2026.
Additional sources	ISSVA classification documents, VASCERN-VASCA patient pathways, GeneReviews chapters, the *Great Ormond Street Handbook of Paediatric Vascular Anomalies*, and reference lists of key reviews and disease-specific studies.
Core search terms	"vascular malformation", "vascular anomaly", "extremity", "pediatric orthopaedics", "venous malformation", "lymphatic malformation", "arteriovenous malformation", "fibro-adipose vascular anomaly", "Klippel-Trenaunay", "Parkes Weber", "capillary malformation arteriovenous malformation", "PIK3CA-related overgrowth spectrum", "limb length discrepancy", "epiphysiodesis", "localized intravascular coagulopathy", "sirolimus", "alpelisib", and "trametinib".
Inclusion focus	Pediatric and adolescent extremity vascular malformations; musculoskeletal involvement; pain, contracture, joint involvement, LLD, overgrowth, gait disturbance, peri-procedural coagulation risk, imaging, referral, interventional treatment, surgery, rehabilitation, systemic therapy, and targeted therapy.
Exclusion focus	Studies unrelated to extremity disease, adult-only nonorthopaedic studies without transferable clinical relevance, purely technical interventional reports without implications for pediatric orthopaedic management, and vascular tumor literature not relevant to differential diagnosis or diagnostic safety.
Evidence priority	Official classification documents and specialist pathways were prioritized, followed by pediatric/multidisciplinary reviews, disease-specific cohort studies and prospective studies, surgical series, outcome-measurement studies, and standard pediatric orthopaedic reviews or consensus documents when disease-specific evidence was unavailable.
Pediatric evidence strategy	Pediatric and adolescent data were prioritized whenever available. Adult or mixed-age data were used only when pediatric extremity-specific evidence was limited and were interpreted cautiously.
Orthopaedic extrapolation	LLD thresholds, epiphysiodesis indications, and follow-up intervals were derived from general pediatric orthopaedic literature and are presented as pragmatic guidance rather than vascular-malformation-specific prospective evidence.
Outcome-measurement evidence	Peripheral vascular malformation outcome studies and quality-of-life literature were included to support discussion of patient-centered and limb-centered endpoints. Adult PROM data were not treated as directly equivalent to pediatric orthopaedic evidence.
Screening process	Articles were selected by clinical relevance and evidence level. Formal duplicate independent screening and inter-reviewer adjudication were not performed because this was a narrative review rather than a systematic review.
